# Natural Compounds Targeting MAPK, PI3K/Akt, and JAK/STAT Signaling in Papillary Thyroid Cancer

**DOI:** 10.3390/ijms262110498

**Published:** 2025-10-29

**Authors:** Michelle Carnazza, Nan Yang, Raj K. Tiwari, Jan Geliebter, Xiu-Min Li

**Affiliations:** 1 Division of R&D, General Nutraceutical Technology LLC, Briarcliff Manor, NY 10510, USA; michelle.carnazza@gnt-us.com (M.C.); nan.yang@gnt-us.com (N.Y.); 2 Department of Pathology, Microbiology & Immunology, New York Medical College, Valhalla, NY 10595, USA; raj_tiwari@nymc.edu (R.K.T.); xiumin_li@nymc.edu (X.-M.L.); 3 Department of Otolaryngology, New York Medical College, Valhalla, NY 10595, USA; 4 Department of Dermatology, New York Medical College, Valhalla, NY 10595, USA

**Keywords:** papillary thyroid cancer, natural compounds, traditional Chinese medicine

## Abstract

Thyroid cancer (TC) represents the most prevalent endocrine malignancy, with papillary thyroid cancer (PTC) comprising approximately 80% of cases and accounting for the majority of annual incidence and mortality. PTC is generally confined to the thyroid gland and demonstrates an excellent prognosis after surgery, with a five-year survival rate exceeding 90%. Nevertheless, recurrence can occur, and the ten-year survival rate for the advanced PTC is below 50%. Even after effective successful surgical intervention, many still require ongoing surveillance, additional treatment, and lifelong thyroid hormone replacement, while facing the potential adverse effects such as hormone fluctuations, surgical complications, and sequelae of radioactive iodine exposure. Naturally occurring compounds have demonstrated anti-cancer properties and hence the potential to be used as therapeutic options, impacting the same drivers and pathways involved in the tumorigenesis of thyroid cancer. This narrative review focuses on the natural compounds’ convergence on molecular nodes of PI3K/Akt, MAPK, and JAK/STAT to overcome therapeutic resistance and restore apoptosis, highlighting their potential in advanced and recurrent PTC.

## 1. Introduction

Thyroid cancer (TC) is the frequently diagnosed endocrine malignancy and its incidence in the United States has risen steadily over recent decades, estimated at about ~3% annually [1]. In 2025, there is an estimate of approximately 44,020 new diagnoses and 2290 disease-related deaths [2]. Among females, TC is the 7th most common cancer, accounting for 3% of all cancers, and is the leading cancer type in females 20–29 years old [3,4]. Papillary thyroid cancer (PTC) comprises 80% of TC cases and is responsible for most of the annual morbidity and mortality. While PTC is generally confined to the thyroid gland and demonstrates excellent prognosis, with surgical treatment yielding five-year survival rate exceeding 90% following surgery, recurrence can occur [5]. In advanced PTC, the ten-year disease-specific survival decreases to less than 50% [6]. 

**Figure 1:**
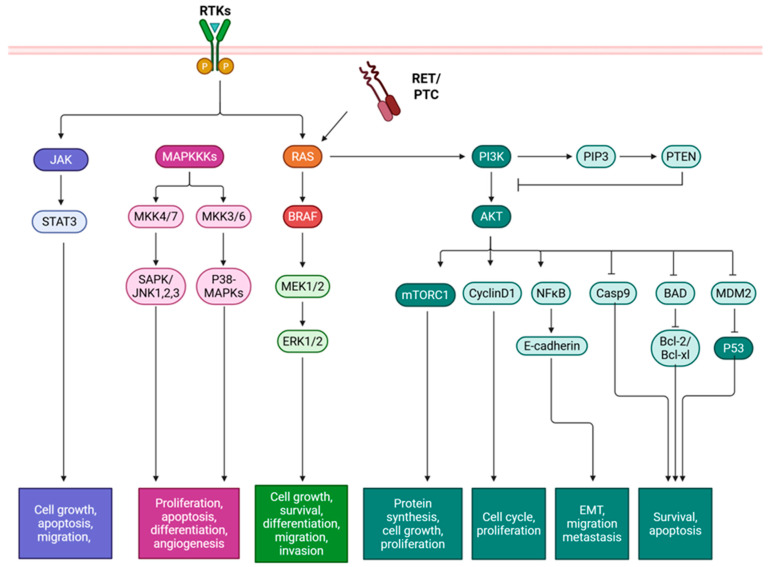
Mutations in papillary thyroid cancer and their effects on downstream signaling pathways. Tyrosine kinase receptor rearrangement RET/PTC and point mutations in BRAF and RAS result in constitutive activation of signaling pathways MAPK, PI3K/Akt, and JAK/STAT, which affect gene expression and subsequently activation cell growth, proliferation, differentiation, migration, invasion, and survival. BAD: Bcl-2-associated agonist of cell death, Bcl-XL: B-cell lymphoma extra-large, Bcl-2: B-cell lymphoma 2, BRAF: B-rapidly accelerated fibrosarcoma, Casp: Caspase, ERK: Extracellular signal-regulated kinase, JAK: Janus kinase, JNK: c-Jun N-terminal kinase, MAPK: Mitogen-activated protein kinase, MKK: MAPK kinase, MDM2: Mouse double minute 2, MEK: Mitogen-activated protein kinase kinase, mTOR: Mammalian target of rapamycin, NF-κB: Nuclear Factor kappa-light-chain-enhancer of activated B cells, PIP3: Phosphatidylinositol 3,4,5-trisphosphate, PI3K/Akt: Phosphatidylinositol 3-kinase/protein kinase C, PTEN: Phosphatase and tensin homolog, p53: Tumor protein p53, RAS: Rat sarcoma virus, RET/PTC: Rearranged during transfection in papillary thyroid cancer, RTK: Receptor tyrosine kinase, SAPK: Stress-activated protein kinase, and STAT: Signal transducer and activator of transcription. Created in BioRender. Geliebter, J. (2025) https://BioRender.com/tk0szej (accessed on 9 August 2025).

The indolent nature of PTC may be attributable to its comparatively low somatic mutation burden. Although the genomic profile is generally stable, aggressive PTC may occur via de-differentiation, and thus reduce survival [7]. PTC driver mutations result in the constitutive activation of the Mitogen-activated protein kinase (MAPK), phosphatidylinositol 3-kinase/protein kinase C (PI3K/Akt), and Janus kinase (JAK)/Signal transducer and activator of transcription (STAT) pathways. These driver mutations were found to be “mutually exclusive”, as their functions are redundant [8]. Key driver mutations in PTC include B-rapidly accelerated fibrosarcoma (BRAF), rat sarcoma virus (RAS) (N/K/H isoforms), and telomerase reverse transcriptase (TERT) mutations [9]. Additionally, gene fusions of tyrosine kinases Rearranged during Transfection in PTC (RET/PTC), neurotrophic tyrosine receptor kinase (NTRK) 1 and 3 can occur, as well as rearrangement of paired box 8/peroxisome proliferator-activated receptor gamma-1 (PAX8/PPARϒ) have been identified [9]. Of all, the most common driver mutation is the BRAF point mutation, harbored by approximately 75% of TC patients [10]. Of these patients, roughly 67% are the V600E mutation, which correlates with advanced clinical staging and increased lymph node metastasis [7,10]. Another highly prevalent driver mutation, affecting 10–30% of PTC patients, is the gene fusion of RET, which functions in normal developmental cell growth and proliferation. Alterations leading to its activation can lead to cancer, including breast, gastrointestinal, and PTC [11]. The rearrangement that serves as a driver in PTC is termed RET/PTC, however, over 19 different rearrangements have been described, allowing for constitutive activation of RET, and subsequently uncontrolled proliferation and survival [12]. Consequently, as these genetic alterations accrue with other molecular alterations, and MAPK and PI3K/Akt signaling intensifies, it is that the poorly differentiated thyroid cancer and anaplastic thyroid cancer (ATC) can develop [7]. The MAPK and PI3K/Akt pathways’ activation results in downstream signaling of molecules including BRAF, Mitogen-activated protein kinase kinase (MEK), Akt, and Mammalian target of rapamycin (mTOR) [13] (Figure 1). Together, these promote recurrence and increased mortality through promotion of tumorigenesis and activation of cell growth and proliferation, invasion and metastasis.

Diagnosis of PTC is typically established through neck ultrasound and fine needle aspiration, after which staging and risk stratification inform prognosis and guide therapeutics. Standard therapeutic approaches include surgery (lobectomy or total thyroidectomy), radioactive iodine (RAI) ablation of remnant thyroid tissue, thyroid stimulating hormone suppression, external beam radiation, and kinase inhibitors [14,15]. Total thyroidectomy remains the most effective therapeutic, whereas lobectomy, the removal of the affected lobe, may suffice in some cases and does not require lifelong hormone replacement therapy. There have also been trends away from routine RAI for remnant ablation after total thyroidectomy, unless patients are intermediate to high-risk or present with distant metastasis [16]. RAI ablation is orally administered and exploits thyroid follicular cells’ avidity for iodine to eradicate the residual thyroid tissue. While generally well-tolerated, its efficacy is limited, as nearly 50% of persistent, recurrent, or metastatic lesions lose iodine avidity through de-differentiation and downregulation of sodium iodide symporter (NIS) or thyroid peroxidase expression, leading to RR-PTC (radioiodine-refractory papillary thyroid cancer) [17,18]. Additionally, RR-PTC can be treated with tyrosine kinase inhibitors (TKIs), however significant side effects and acquired resistance can occur [16,19,20]. TKIs include those that target RET/PTC, vascular endothelial growth factor receptor (VEGFR), fibroblast growth factor receptor (FGFR), platelet derived growth factor receptor (PDGFR), and proto-oncogene c-Kit (KIT). Lenvatinib, which targets VEGFR1-3, FGFR, RET, PDGFRa, and KIT [21], and Sorafenib, which inhibits VEGFR, PDGFR, RET, KIT, and also serine/threonine kinase RAF [22], are currently approved for progressive RR-PTC [16,23]. Multimodal approaches can lower the risk of persistent or recurrent disease, and metastatic spread, while limiting treatment-related morbidity, including post-operative TSH suppression and central neck dissection, which offers the most benefit to only high-risk patients [24,25]. Ultimately, even after successful surgery, long-term surveillance is required with continued treatment, and lifelong thyroid hormone supplementation, alongside potential adverse effects including hormone fluctuations, surgical complications, and sequelae of radioactive iodine ablation [26]. 

Targeting the MAPK, PI3K/Akt, and JAK/STAT pathways with novel treatments, including herbal medicine, has been suggested [27]. The pleiotropic capabilities of traditional Chinese medicine come from the combination of herbs with many effects and targets. Identification of active compounds enables the focus on specific disease targets. Naturally occurring compounds have demonstrated anti-cancer properties and hence the potential to be used as therapeutic options in diseases like diabetes, asthma, and cancer. Frequently, these compounds will impact the same targets and pathways involved in the tumorigenesis of thyroid cancer [27]. Natural compounds have demonstrated long-lasting effectiveness that is safe and able to regulate multiple targets at once [28]. Natural compounds have been assessed in vitro in PTC cell lines with differing characteristics and mutation statuses (Table 1), to alter these cancer-promoting pathways. Additionally, these ingredients provide advantages to western medicine being more readily available, more convenient, and affordable [28]. This comprehensive review proposes that naturally occurring compounds act as multi-targeted regulators of PTC, with anti-tumor effects that converge on the modulation of MAPK, PI3K/Akt, and JAK/STAT, thereby identifying shared molecular regulators and translational opportunities for integrative therapeutic strategies in advanced or recurrent PTC. 

## 2. Molecular Targets of Natural Compounds in PTC

The naturally occurring compounds have overlapping mechanisms of action that make them potent anti-thyroid cancer mediators alone or in combination with other compounds or current targeted therapies. The natural compounds have great pleiotropy and can affect more than one mechanism of PTC recurrence and resistance. Here, they are organized by primary mechanistic focus, but their pleiotropic effects are also described in Table 2. Many naturally occurring compounds have direct preclinical experimental validation in PTC, while others have broad thyroid cancer and other cancer evidence that can be extrapolated to driver PTC pathways. This highlights their potential translational benefits in PTC, including overcoming acquired resistance, induction of apoptosis or cell cycle arrest, prevention of metastasis, and regulation of oxidative stress and metabolism. 

### 2.1. Overcoming Therapeutic Resistance

#### 2.1.1. Curcumin

Curcumin (C_48_H_28_O_30_; 1,7-bis(4-hydroxy-3-methoxyphenyl)-1,6-heptadiene-3,5-dione), a bioactive, bright yellow-orange polyphenol, is produced by the *Cucruma longa* species, turmeric, which is a member of the ginger family and is traditionally been used as a medicinal herb in Asian countries for its antioxidant, anti-inflammatory, anti-microbial, and anti-cancer properties, including breast, lung, pancreatic, gastric, and brain cancer [35,36]. There is evidence of anti-proliferative and apoptosis inducing effects of curcumin through targeting multiple signaling molecules [37]. In PTC, curcumin has been investigated for its effects on metastatic phenotypes of K1 and BCPAP PTC cell lines. There was a dose-dependent (12.5 μM, 25 μM, 50 μM, 100 μM) suppression on K1 cell viability, attachment, spreading, migration, and invasion, which included a downregulation of matrix metalloprotease (MMP)-9 expression and activity at 50uM [38]. In another study, the proliferative and invasive ability of TPC-1 cells were inhibited in a dose dependent manner (10 μM and 20 μM) through inactivation of JAK2 and STAT3 [39]. In TPC1, this was characterized by cell cycle arrest in G1/G0 and 35% apoptosis with decreased B-cell lymphoma 2 (Bcl-2) expression at 10 and 20 μM. In an additional study, the BCPAP and TPC-1 cells treated with curcumin also showed dose-dependent reduction in cell viability (10 μM 20 μM, 40 μM, 80 μM) mediated by cleavage of caspase-3 [40]. The STAT3 inactivation regulated stemness and apoptosis induced by curcumin at 20 μM [40]. Importantly, this study elucidated a synergistic effect with cisplatin in enhancing apoptosis through JAK/STAT signaling, highlighting curcumin’s potential to be combined with chemotherapy to improve therapeutic outcomes [40]. Confirmation of STAT3-mediated mechanisms of curcumin was observed, with inhibition of invasion, migration, and epithelial-mesenchymal transition (EMT) at cell viability IC50 in TPC1 and BCPAP, at 23.3 μM and 26.4 μM, respectively. The STAT-3 dependent mechanism was identified by modulation of noncoding microRNA (miRNA) miR-301a-3p as a regulator of this axis, and confirming its effects on EMT markers and JAK/STAT activation [41]. Similarly, long noncoding RNA (lncRNA) LINC00691 was inhibited by curcumin treatment of BCPAP cells in a dose-dependent manner (2.5 μM, 5 μM, 10 μM, 20 μM), which attenuated apoptosis and suppressed proliferation and the Warburg effect, mediated by Akt signaling [42]. As hypoxia is an important factor in clinical thyroid cancer therapy, curcumin was tested for and demonstrated dose-dependent (12.5 μM, 25 μM, 50 μM) inhibition of hypoxia-induced reactive oxygen species (ROS), migration under hypoxic conditions, and decreased expression of hypoxia-inducible factor (HIF)-1α at both the RNA and protein level [43]. Curcumin was able to decrease the HIF-1ꭤ binding to hypoxia response element [43]. Similar results were obtained in BCPAP, whereby curcumin treatment reduced cell viability, attachment, spreading and migration in a dose dependent manner (12.5 μM, 25 μM, 50 μM) [37]. These results were accompanied by a dose-dependent increase in E-cadherin expression and decrease in vimentin, MMP-2, and MMP-9 expression and reductions in transforming growth factor (TGF)-β1-mediated phosphorylation of Smad2 and Smad3 [37]. Autophagy was also markedly induced by curcumin treatment of TC cells, increasing microtubule associated protein 1A/1B light chain (LC3)-II conversion, beclin-1 accumulation, p62 degradation, and acidic vesicular organelle formation [44]. These effects were specific to thyroid cancer cells, without affecting normal thyroid epithelial cells [44]. 

Curcumin of various formulations and doses have been assessed in Phase I clinical trials of small samples size, short duration, and across various cancer stages and populations, making it difficult to strongly recommend it as a cancer treatment [45]. The efficacy of curcumin on malignant diseases overall has not been substantiated by the current available studies. [46]. Curcumin has not specifically undergone a clinical trial for papillary thyroid cancer treatment, however a nanoparticle formulation containing curcumin (40 and 80 mg) as a therapeutic supplement showed a protective effect against RAI oral complications, in a double blind, placebo-controlled clinical trial of 90 patients [47]. Curcumin is a well-known pleiotropic modulator of signaling pathways, including those of PTC. Its low oral bioavailability has been improved with nanomedicine, and in combination with its high safety and synergistic potential, makes curcumin a promising PTC therapeutic strategy [48,49].

#### 2.1.2. Quercetin

Quercetin is one of the most widely used bioflavonoids for the treatment of metabolic and inflammatory disorders [50]. Quercetin (C_15_H_10_O_7_; 3,3′,4′,5,7-pentahydroxyflavone) is a bioactive flavonoid present in plants, found mostly in onions, grapes, berries, cherries, broccoli, and citrus fruits [50]. Flavonoids give many fruits, flowers, and vegetables their colors and are potent antioxidants [50]. Therefore, their anti-cancer properties have been investigated in blood, prostate, and lung cancer [51]. In PTC, quercetin dose-dependently (10 μM, 50 μM) increased BCPAP cell permeability and decreased mitochondrial membrane potential, and inducing cell cycle arrest at the S phase, indicating an induction of apoptosis [52]. Quercetin induced pro- non-steroidal anti-inflammatory drug activated gene (NAG)-1 expression, but not mature NAG-1, mediated by promoter activation by transcription factor CCAAT/enhancer-binding protein [52]. Quercetin treatment from 10–200 μM over 72 h showed both inhibitory and proliferative effects depending on dose and time. Quercetin at 50 μM and 75 μM for 24 h, showed cell viability decreased and the rate of apoptosis increased through mechanisms including caspase activation and reduction of heat shock protein-90 expression [53]. The less pronounced effect on cell viability after 24 h was owed to the proliferative effect and short half-life of quercetin [53]. In BCPAP cells, cell adhesion, migration, and EMT markers were modulated (reduced MMP-9 and increased E-cadherin) while NIS mRNA levels and radioiodine uptake were increased when treated with 100 μM of quercetin for 24 h [54]. This suggested that quercetin could be useful as an adjuvant for RAI. Further extrapolation of the mechanism of quercetin-mediated reduction in PTC viability highlighted a reduction in glycolysis, as evident through the reduction in extracellular acidification rate, that is required for cell migration [55]. This was evident when treated with quercetin at 20 μM, 40 μM, 60 μM, or 80 μM, for 48 h. Reduction in cell migration and serine-39 phosphorylation of vimentin upon quercetin treatment were observed at much lower doses, including 2.5 μM, 5 μM, and 10 μM [55]. Furthermore, validation of intrinsic apoptosis and activation of caspase-3 through extracellular related kinase (ERK) was demonstrated along with a reduction in the cancer stem cell population of the PTC cells [55]. These outcomes indicate that a downstream effects mediated by Akt and ERK activation of caspase-3 [55]. A bioinformatic analysis of potential targets or quercetin was performed and molecular docking data showed that the binding capacity of quercetin with PTC was significant in the key targets of MMP-9, Jun proto-oncogene (JUN), osteopontin, and heme oxygenase 1 (HMOX1) [56]. These were verified with in vitro experiments demonstrating quercetin treatment at 200 μM not only reduced MMP-9, JUN, and HMOX1 expression, but significantly suppressed cell proliferation, migration, and invasion of K1 and TPC-1 cells while increasing apoptosis [56]. Quercetin was identified as a core component in *Prunella vulgaris* L. treatment of PTC, with a high binding affinity for tumor protein 53 (TP53) in silico and in vitro dose-dependent reduction in BCPAP cell viability (2.5 μM, 5 μM, 10 μM, 20 μM, 40 μM, 80 μM, 160 μM) [57]. Another network pharmacology study highlighted quercetin as a thyroid cancer treatment, with high binding affinity to modulators including MMP-1, C-X-C motif chemokine ligand-8, MMP-3, and collagen type 1 ꭤ1 [58]. At 400 uM, quercetin reduced thyroid cancer cell viability, MMP-1 and MMP-3 expression in vitro [58]. These data demonstrate that quercetin regulates a variety of proteins and signaling pathways in PTC underlying its anti-cancer effects. 

Quercetin is a powerful anti-inflammatory compound with antioxidant properties and strong in silico and in vitro evidence for its used in combination therapy. It has not been assessed in clinical trials of PTC for its use as a cancer therapy alone or in combination. Its low toxicity (up to 1000 mg daily) and synergistic potential indicate investigation into its beneficial concentration threshold in PTC may elucidate its therapeutic potential and clinical readiness as an oral supplement (with daily intake ranging from 10–500 mg) [59]. Due to its hydrophobic structure, quercetin demonstrates low bioavailability [60], therefore improvements in its formulation and administration will be necessary for its use as an effective therapeutic. 

#### 2.1.3. Luteolin

Luteolin (C_15_H_10_O_6_; 3′4′5,7-tetrahydroxyflavone) is a flavonoid, which is very prevalent in TCM, with diverse pharmacological actions, including anti-inflammatory, anti-diabetic, antioxidant, and anti-cancer [61]. Luteolin regulates several cancer signaling pathways including apoptosis, angiogenesis, and metastasis, in various cancer types including colon, lung, prostate, gastric, liver, and breast cancer [61]. *Prunella vulgaris* (PV) has demonstrated anti-PTC effects in TPC-1, time- and dose-dependently reducing cell viability (5, 10, 20, 30% (*v*/*v*)) [62]. At the IC50 of 16.3% PV, apoptotic morphology was induced including small size, round shape, detachment, chromatin condensation, and nuclear fragmentation that coincided with induction of DNA fragmentation and apoptosis-associated proteins Bcl-2, Bcl-2 associated X protein (Bax), and caspase-3 [62].

As with quercetin, luteolin was identified as a core component of *Prunella vulgaris* L. for treatment of PTC [57]. Luteolin demonstrated in silico high binding affinity for TP53 and in vitro dose-dependent reduction in BCPAP cell viability (2.5 μM, 5 μM, 10 μM, 20 μM, 40 μM, 80 μM, and 160 μM) [57]. In another screening of flavonoids for their effect on NPA cells proliferation, luteolin was identified, with IC50 ranging from 21 μM to 32 μM [63], Investigation into the molecular targets of luteolin demonstrated a dose-dependent reduction in IHH-4 cell viability (10 μM, 20 μM, 40 μM, and 80 μM) [64]. At 10 μM, luteolin reduced thyroid cancer clonogenicity and increased proportion of cells in the G0/G1 phase of the cell cycle [64]. Mechanistically, this was determined to be due to the downregulation of lncRNA BRAF-activated non-protein coding RNA and the subsequent reduction in thyroid stimulating hormone receptor (TSHR) causing downstream reduction in cyclic adenosine monophosphate (cAMP)/protein kinase A/cAMP-responsive element-binding protein and cyclin D1 that prevents tumor growth downstream of Akt [64,65]. This was confirmed in a xenograft model of thyroid cancer, whereby luteolin treatment at 50 mg/kg decreased tumor growth in vivo, and this anti-tumor effect was abolished by BANCR overexpression [64]. 

Sorafenib is an food and drug administration-approved treatment for advanced, RR-PTC [66]. While not investigated in PTC, luteolin shows synergistic effects with sorafenib in apoptosis induction of hepatocellular carcinoma [67], warranting its investigation as a synergistic compound in sorafenib-treated PTC. A major limitation of luteolin as an anti-PTC therapy comes from its low bioavailability in vivo, due to its poor stability and low absorption rate [68]. Enhancement of bioavailability by various drug delivery strategies has been employed but not investigated in thyroid cancer. In other types of cancer, luteolin has been suggested as a synergistic therapy with chemotherapy-resistance [69]. No clinical trials have specifically investigated the use of luteolin for papillary thyroid cancer treatment. While preclinical and in vivo studies have validated the safety of luteolin [61] clinical trials will be necessary to support its clinical development [68].

#### 2.1.4. 3,3′-diindolylmethane (DIM)

DIM (C_17_H_14_N_2_; 3,3’-diindolylmethane) is a heterocyclic and bioactive dimer derived from the endogenous conversion of indole-3-carbinol (I3C) [70]. I3C is a naturally occurring glucosinolate found in a wide variety of plant food substances, including cruciferous vegetables, such as broccoli, cabbage, cauliflower, and brussels sprouts [71]. DIM has proven to be very beneficial due to its antioxidant, anti-estrogenic, and anti-cancer activities, including prostate and breast cancer [71]. In vitro, DIM demonstrated anti-proliferative activity in BCPAP at 50 μM that was mediated by a G1 arrest with the induction of apoptosis [72]. DIM treatment at 25 μM also was shown to reduce angiogenesis induced by BCPAP [73]. The same group demonstrated BCPAP treated with 25 μM DIM resulted in reduced estrogen-mediated migration, adhesion and invasion, specifically through reductions in MMP-2 and MMP-9 [74]. In their Phase 1 clinical trial study of patients with thyroid proliferative disease (TPD), encompassing any abnormal growth of thyroid cells, benign or malignant, and DIM treatment (300 mg) enhanced the degradative metabolism of estrogen and therefore through this anti-estrogenic activity could serve as a supplement to reduce the risk of TPD development [75]. In breast cancer, this phenomenon was mediated by inhibition of Akt [76]. Similarly, in their study of mis-identified thyroid originating cell line KAT50-TS, determined to be a colorectal cancer cell line, DIM inhibited cancer growth in a dose-dependent manner (25 μM, 50 μM, 75 μM, 100 μM, and 500 μM) and inhibited adhesion, migration, and invasion (25 μM and 50 μM) [77]. This was mediated by downregulation of Akt and ERK activation and subsequent downregulation of G1-S cell cycle markers cyclin D1, cyclin-dependent kinase (cdk)-6, and cdk4, upon treatment with 25 μM and 50 μM of DIM [77]. This suggests that DIM is an attractive hormone-related cancer treatment option that demonstrates synergistic effects with kinase inhibitors. 

DIM has demonstrated very low oral bioavailability owed to its poor solubility in physiological liquids, emphasizing the challenges for its use as a therapeutic or supplement. Drug delivery formulations have been developed to overcome this limitation, including nano-formulation. Nano-formulated DIM (DIM-PLGA-PEG/chitosan NP) dose-dependently reduced breast cancer cell viability at 50, 10, 150, and 200 μg/mL and reduced migration and induced apoptosis at lower concentrations, including 12.5, 6.25, 3.125 μg/mL [78]). At 5 μg/mL. Nano-formulated DIM reduced angiogenesis in chick embryos [78]. This was elucidated in silico with molecular docking predicting binding to Bax, Bcl-2, and p53 binding sites and domains [78]. DIM fermentation and boiling also sought to enhance DIM concentration in cabbage, and upon in vivo treatment in mice, ameliorated doxorubicin cardiotoxic effects [79]. In a clinical trial of breast cancer patients, oral BioResponse-DIM demonstrated an excellent safety profile at both 75 mg and 150 mg twice daily and favorable effects on estrogen metabolism [80]. However, DIM also reduced tamoxifen metabolites when co-administered, which presumptively are responsible for tamoxifen efficacy, and therefore, needs to be studied further before recommending DIM supplementation with patients receiving tamoxifen [80]. Therefore, optimization of DIM has synergistic therapeutic potential in papillary thyroid cancer. 

#### 2.1.5. Aloperine

Aloperine (C_15_H_24_N_2_; (1R,2S,9R,10R)-3,15-diazatetracyclo[7.7.1.02,7.010,15]heptadec-7-ene) is a quinolizidine alkaloid that is isolated from *Sophora alopecuroides* seeds and leaves [81]. Aloperine has demonstrated therapeutic effects against various pathologies due to its antioxidant, anti-inflammatory, anti-allergic, antiviral, and anti-cancer activities, in cancer types including colon, pancreatic, breast, liver, osteosarcoma, multiple myeloma, and glioma [81]. Aloperine treatment of human multi-drug resistant PTC cells, IHH-4, demonstrated reduced cell viability at 24, 48, and 72 h of treatment at doses over 200 μM. Aloperine inhibited of colony formation at as low as 50 μM and tumorigenesis at 200 μM, with the promotion of cellular apoptosis, evident through the activation of caspase-8, -9 and -3, and poly (ADP-ribose) (PARP) [82]. In another study, IHH-4 cells treated with aloperine demonstrated reduction in cell viability at concentrations over 200 μM through autophagy activation, evident by LC3-II expression and inhibition of the Akt signaling pathway [83].

There is no evidence for the use of aloperine in thyroid cancer beyond in vitro and in vivo investigation, urging the necessity for clinical trials. The pharmacokinetics of alopereine also required further investigation to promote drug development, with bioavailability in rat plasma around 45% [84]. Safety and dosage issues are also a concern of aloperine, as it is considered harmful if ingested, leading to hepatotoxicity and nephrotoxicity in mice in vivo [85].

### 2.2. Apoptosis and Cell Cycle Arrest

#### 2.2.1. Berberine

Berberine (BBR; C_20_H_18_NO_4_^+^; 16,17-dimethoxy-5,7-dioxa-13-azoniapentacyclo[11.8.0.02,10.04,8.015,20]henicosa-1(13),2,4(8),9,14,16,18,20-octaene) is an isolquinoline alkaloid purified from Chinese herbs [86]. This naturally occurring compound is crystal bright yellow in color and present in different parts such as roots, stem, bark, rhizome, fruit and leaves of several plant species [86]. BBR demonstrates antioxidant, anti-inflammatory, anti-diabetic, antibacterial, anti-obesity, and anti-cancer effects, including myeloma, gastric, lung, and colon cancer [87]. The anti-cancer effects of BBR mainly include apoptosis, cell cycle arrest, autophagy, and inhibition of metastasis [87]. In PTC, treatment with BBR inhibited cell growth of TPC-1 cells in a dose-dependent manner at 1, 10, or 100 μM for 72 h [88]. This was demonstrated to be caused by cell cycle arrest in the G0/G1 phase and upregulation of p27 at 10 μM [88]. Another study also demonstrated that BBR inhibited TPC-1 proliferation starting at 10 μM and induced apoptosis in a dose- and time- dependent manner [89]. BBR-treated TPC-1 cells were arrested in the G0/G1 phase with an upregulation in p21 and downregulation of cyclin E1 and cdk2 at 20 μM and 80 μM [89]. An increase in cleaved caspase-3 and ratio of Bax:Bcl-2 is consistent with the induction of mitochondrial apoptosis [89]. This coincided with the loss of mitochondrial membrane potential and impairment of mitochondrial functions. BBR treatment also inhibited migration of TPC-1 cells whereby expression of vimentin, p-Akt1, p-ERK, and p-c-Jun N-terminal kinase (JNK) were decreased [89]. Similarly, in K1 cells, BBR treatment inhibited K1 cell proliferation, which was associated with reduced PI3K/Akt signaling activation and the reduced expression of nuclear factor erythroid 2-related factor 2 (Nrf2) and its nuclear translocation [90]. This was confirmed in another study of TPC-1 and K1 treated with 20, 40, or 80 μM of BBR showing dose-dependent reductions in cell viability and the induction of apoptosis at 40 μM [91]. This was further elucidated by the reduction of Bcl-2 and induction of cleaved caspases three and nine [91]. In xenograft models, the authors demonstrated BBR inhibits tumor growth in xenograft and transgenic mouse models at 100 mg/kg and 200 mg/kg [91]. Mechanistically, the authors concluded BBR inhibits Nrf2-depdendent PI3K/Akt signaling and results in ROS production [91].

Hindering the ability to use berberine for its metabolic regulation and tumor suppressing mechanisms is its extremely low bioavailability of less than 1% [92]. Berberine has, however, been deemed safe at recommended doses and therefore nanoformulations and other methods to enhance bioavailability have been developed [92,93]. Clinical trials to evaluate the effectiveness of enhanced BBR systems are still needed. Clinical trials to evaluate the effect of BBR in papillary thyroid cancer are also needed for the development of its clinical applications.

#### 2.2.2. Myricetin

Myricetin (C_15_H_10_O_8_; 3,5,7-Trihydroxy-2-(3,4,5-trihydroxyphenyl)-4H-chromen-4-one) is a polyphenol of the flavonoid class that is commonly sourced from vegetables (including tomatoes), fruits (including oranges), nuts, berries, tea, and red wine and is used as a preservative agent in foods and beverages containing oils and fats. Myricetin has demonstrated neuroprotective, anti-diabetic, antioxidant, and anti-cancer properties, including in breast, gastric, and ovarian cancer [94]. Treatment of PTC cells (SNU-790) with myricetin resulted in a dose-dependent cytotoxic effect and the induction of DNA condensation at 50 and 100 μM [95]. Evidence supported that an apoptotic mechanism causing these effects through activation of caspase cascades, upregulation of the Bax:Bcl-2 expression ratio, the induction of apoptosis-inducing factor, and the alteration of the mitochondrial membrane potential [95]. In the human colon cancer cells, HT-29, HCT116, SW480 and SW620, myricetin induced apoptosis at 50 and 100 μM within 48 h, by inhibition of PI3k/Akt signaling. Whether myricetin induces apoptosis in PTC by a similar mechanism remains to be determined, and therefore this may be the same mechanism in PTC [96].

Myricetin has low oral bioavailability, at around 10% and has a generally good safety profile at recommended dietary doses with an LD50 of 800 mg/kg in mice [97]. The toxicity seen against cancer cell lines spares the normal cells in vivo. [98,99]. Nanoformulations of myricetin enhance anti-tumor activity in vivo [100], but this has not specifically been investigated in PTC. Clinical trials establishing the anti-PTC mechanisms of myricetin have yet to be conducted.

#### 2.2.3. Punicalagin

Punicalagin (C_48_H_28_O_30_; D-Glucose, cyclic 4,6-(2,2’-(5,10-dihydro-2,3,7,8-tetrahydroxy-5,10-dioxo(1)benzopyrano(5,4,3-cde)(1)benzopyran-1,6-diyl)bis(3,4,5-trihydroxybenzoate)) cyclic 2,3-(4,4’,5,5’,6,6’-hexahydroxy(1,1’-biphenyl)-2,2’-dicarboxylate)-, (2(S),4(S,S))-) is a phenolic compound that is derived from the peel of the pomegranate, *Punica granatum*. Punicalagin is converted by the human colonic microbiota to ellagic acid and then to a urolithin A derivative and has demonstrated beneficial characteristics including hepatoprotective, antioxidant, anti-inflammatory, and anti-cancer properties, including in cervical, breast, ovarian, lung, and colorectal cancer [101]. When investigated for its effects on PTC, punicalagin treatment at 50 μM and 100 μM reduced the viability of BCPAP cells through the induction of autophagy, demonstrated through the lack of nuclear fragmentation, chromatin condensation, caspase-3 and PARP cleavage and the induction of LC3-II conversion and beclin1 expression [102]. Autophagy was promoted by punicalagin treatment through MAPK activation and mTOR inhibition [102]. Autophagic cell death induced in another cell line, BCPAP was characterized by increased H2A.X phosphorylation, causing DNA breaks and triggering of an Ataxia-telangiectasia Mutated-mediated DNA damage response at 50 μM and 100 μM of punicalagin [103].

In addition to autophagy, punicalagin induced senescence in BCPAP cells at 100 μM [104]. Senescence is characterized by an altered morphology of cell granularity that is accompanied by senescence-associated beta-galactosidase staining, which was evident in punicalagin-treated BCPAP cells [104]. In this study, this cell cycle arrest coincided with the upregulation of p21 and a SASP (senescence-associated secretory phenotype), characterized by high levels of inflammatory cytokines, mainly interleukin (IL)-6 and IL-1β [104]. Senescent growth arrest and SASP were triggered by Nuclear Factor kappa-light-chain-enhancer of activated B cells (NF-κB) activation, as demonstrated through the phosphorylation and subsequent degradation of IκBα as well as the nuclear translocation of p65 [104].

Punicalagin is characterized by low oral bioavailability (3–6%) [105] and no toxicity, even at high oral doses in vivo [106,107]. Punicalagin has not been investigated in clinical trials for its effects on PTC but has been investigated for its effects on the microbiome at 75 mg per day [108].

#### 2.2.4. Sanguinarine

Sanguinarine (C_20_H_14_NO_4_^+^; 13-methyl (1,3) benzodioxolo (5,6-c)-1,3-dioxolo (4,5) phenanthridinium) is a bitter crystalline benzophenathridine alkaloid derived from the root of *Sanguinaria canadensis* and other poppy-fumaria species of Papaveraceae family [86,109]. Sanguinarine has demonstrated a variety of properties including antioxidant, antimicrobial, antifungal, anti-inflammatory, antihypertensive, and anticancer, including breast, cervical, ovarian, liver, lung, gastric, and colorectal cancer [110,111]. The investigation into the anti-cancer effects in PTC, revealed that sanguinarine treatment significantly inhibited cell proliferation of PTC cells BCPAP and TPC-1 in a dose and time-dependent manner through apoptosis and autophagy signaling cascades at 4 μM, including p-STAT3, caspase-3, caspase-8, PARP cleavage, and the generation of reactive oxygen species [112]. PTC cytotoxicity was evident in both cell lines at 2 μM, 4 μM and 8 μM after 24 h of treatment [112]. Further, sanguinarine treatment sensitized TPC-1 cells to cisplatin, a chemotherapy drug, at 2 μM and suppressed the growth of PTC thyroid spheroids with a downregulation of stemness markers ALDH2 and SOX2 [112].

While the preclinical data shows promise for the anti-PTC effects of sanguinarine, there are no active clinical trials, highlighting the need for more preclinical evidence. Sanguinarine has shown great potential in drug design that has been heightened by drug delivery methods, however further investigation is needed [110]. Sanguinarine has also been assessed for acute toxicity via oral, intravenous, and dermal routes showing LD50s of 1658, 29, and >200 mg/kg, respectively across rats and rabbits, but translatability to humans is still necessary for the next step of clinical development [86,113].

#### 2.2.5. Genistein

Genistein (C_15_H_10_O_5_; 4′,5,7-Trihydroxyisoflavone) was first isolated from the brooming plant Dyer’s Genista tinctoria L. and is widely distributed in the Fabaceae family, consisting of dietary legumes such as soybean and fava bean [114]. This isoflavone is structurally similar to estrogen and exhibits estrogen-like functions [114]. Genistein has demonstrated anti-inflammatory, antioxidant, antibacterial, antiviral, and anti-cancer activities, including in liver, gastric, lung, colorectal, breast, and anaplastic thyroid cancer [114,115,116]. Genistein has been demonstrated to exert antiproliferative effects on PTC that are not genotoxic, but reduced oxidative-induced DNA damage in primary PTC cells [63,117]. Treatment of PTC cell lines BCPAP, BHP 10-3, and IHH-4 with genistein resulted in significantly reduced proliferation involving arrest in the G2/M phase of the cell cycle [118]. Inhibition of BCPAP and IHH-4 is evident at 40 μg/mL and 80 μg/mL after 72 h of treatment with cell death starting at 10 μg/mL. Modulations in cell cycle markers cyclin B1, D1, and A2 began at as low as 5 μg/mL of genistein treatment. Cell invasion was also decreased at as low as 5 μg/mL and partial reversal of EMT was demonstrated in a dose-dependent manner (5, 10, 20, and 40 μg/mL), with evidence suggesting cytoplasmic translocation of β-catenin being responsible [118].

Clinical trials of genistein elucidate the drawback of low oral bioavailability that results in ambiguous effects and variations in outcomes [119]. Synthetic genistein has demonstrated to be well-tolerated in prostate cancer patients administered 30 mg daily with 100-fold increased plasma concentrations after treatment [120]. Genistein has yet to be evaluated for its effects on PTC in clinical trials, but preclinical studies suggest its investigation would be beneficial.

#### 2.2.6. Capsaicin

Capsaicin (C_18_H_27_NO_3_; (*E*)-*N*-[(4-hydroxy-3-methoxyphenyl) methyl]-8-methylnon-6-enamide)) is a capsaicinoid of the vanilloid family. A major ingredient of red or hot chili pepper and responsible for its pungency, capsaicin has become increasingly attractive due to its antioxidant, anti-inflammatory, analgesic, and anti-cancer effects, including colorectal, breast, prostate, bladder, lung, and nasopharyngeal cancer [121]. Capsaicin-treated BCPAP cells demonstrated a dose-dependent inhibition (25–100 μM) of multiple steps of the metastatic cascade, including migration, invasion and adhesion, without affecting cell viability, due to decreased protein levels of MMP-2, MMP-9, and EMT transcription factors Snail1 and Twist1 and increased E-cadherin expression [122]. The same group observed apoptosis induction by capsaicin in ATC cell lines 8505C and FRO (50–200 μM) and partially restored NIS-mediated RAI uptake at 200 μM [123,124]. Capsaicin targeting of PI3k/Akt, ERK and JNK, as seen in fibrosarcoma cell lines, warrants continued investigation [125].

For oral and gastrointestinal administration of capsaicin, at 30 mg/kg of rat body weight, 50–90% is absorbed with tissue concentration around 25% that decreases to about 1% by 24 h [126], suggesting it is rapidly metabolized in the liver. Nano-formulations of capsaicin have been suggested to enhance the therapeutic effects that otherwise are not seen due to the low bioavailability. For example, in ATC, co-administration of capsaicin and doxorubicin in nano-formulations enhanced therapeutic efficacy [127]. While the consumption of chili peppers would bring about burning that would result in the cessation of consumption before reaching toxic levels of capsaicin, the effective dose that does not affect non-cancerous cells or cause physical discomfort to patients. It has been reported that when capsaicin is administered in combination with other drugs, dose adjustments may be required, as capsaicin can inhibit their intestinal absorption [128]. Conversely, other studies have shown capsaicin pretreatment enhanced bioavailability of cyclosporin in vivo [129], underscoring the importance of rigorous evaluation of drug-drug interactions and synergistic effects on a case-by-case basis. In clinical trials, capsaicin is largely being review for chemotherapy adverse effects [130] and neuropathy [131] as a topical agent, but currently is not being assessed in thyroid cancer.

### 2.3. Anti-Metastasis

#### 2.3.1. Apigenin

Apigenin (C_15_H_10_O_5_; 4′,5,7-trihydroxyflavone) is a flavonoid that is abundant in vegetables, fruits and beverages, such as parsley, grapes, apples, chamomile tea and red wine [132]. Physiologically, it has antioxidant, anti-inflammatory, antibacterial, antiviral, and anti-cancer properties, including breast, cervical, ovarian, pancreatic, colon, liver, esophageal, lung, skin, prostate, and brain cancer [132,133]. In the screening of flavonoids for their effect on NPA proliferation, apigenin was identified, with IC50 ranging from 21 to 3 2 μM [63]. Research into the effects of apigenin on PTC in BCPAP cells demonstrated a dose-dependent decrease in cell viability due to the induction of autophagy, as evident by the accumulation of beclin-1, conversion of LC3 protein, degradation of p62, and formation of acidic vesicular organelles [134]. Furthermore, reactive oxygen species production increased, which resulted in significant DNA damage accumulation of cells in the G2/M phase through the downregulation of cell division cycle 25c expression [134]. Network pharmacological analysis and molecular docking identified core targets of apigenin for PTC treatment including TP53, AKT-1, epidermal growth factor receptor, proto-oncogene tyrosine-protein kinase Src, vascular endothelial growth factor A, and JUN [135]. This strongly suggests that key pathways involved in apigenin treatment for PTC include the regulation of apoptosis and cell proliferation, largely mediated by PI3k-Akt/p53 signaling [135].

Apigenin is structurally similar to luteolin and is also characterized by low toxicity but also low bioavailability. At oral doses ranging from 13.5 to 60 mg/kg, half-life is short, with relative oral bioavailability at about 30% [136]. However, it has been suggested it is possible to reach systemic targets through circulatory circulation of apigenin following oral dosing of its pure form [136]. Apigenin also has implications for negative drug interactions including chemotherapies and medications for supportive cancer care, resulting in their decreased metabolism and toxicity, mediated by inhibition of cytochrome P450 enzymes by apigenin [136]. Much more work is needed to elucidate if apigenin administration can achieve clinically beneficial outcomes with cancer treatment. Clinical trials of apigenin include investigation of neuropsychiatric disorders including Alzheimer’s, anxiety, and insomnia [137]. In the context of cancer, apigenin treatment has not been used outside of animal models, therefore more investigation is required for its use as a therapeutic modality in PTC.

#### 2.3.2. Annurca Flesh Apple Polyphenol Extract (AFPE)

*Annurca* Flesh Apple, also known as the “queen of apples”, is a variety of apple from southern Italy with a white, crisp flesh and a sweet-tart flavor. Apple fruits are one of the most important sources of polyphenolic compounds in the Western diet. *Annurca* Flesh Apple polyphenol extract (AFPE) is a mixture of polyphenolic compounds that has demonstrated many potential health benefits including cardioprotective, antioxidant, anti-arteriosclerosis, anti-hypertensive, and anti-cancer activity, including in breast and colorectal cancer [138,139]. Upon treatment of ATC cell line CAL62 with apigenin, dose dependent (250, 500, 750, 1000 μM) decreases in cell viability and number were observed after 24 h [140]. Treatment of PTC cell line TPC-1 with AFPE (500, 750, 1000 μM) confirmed an accumulation in the G1 cell cycle phase, and a reduction in cell viability with an increase in cell death [140]. TPC-1 cells pretreated with AFPE (500 μM) and subjected to hydrogen peroxide (H_2_O_2_) to induce oxidative stress also experienced an increase in cell death [140], which is significant, as tumorigenesis of the thyroid cells are often associated with oxidative stress. Follicular thyroid cells require H_2_O_2_ for hormone synthesis [141] and excessive production of H_2_O_2_ causes cell transformation and genomic instability. AFPE treatment modulated miRNA expression, including miR-145, which is known to down-regulate Akt [140]. The authors noted that this agreed with AFPE treatment of MDA-MB-231 breast cancer cells, whereby it inhibited AKT activation and downregulated several oncoproteins, such as NF-kB, c-myc, and β-catenin [142].

Evidence suggests that Annurca apple polyphenols are gastric-sensitive or poorly absorbed intestinally, upon oral ingestion starting with the salivary glands [143]. While not a single compound, the whole extract of AFPE used for oral supplementation has been evaluated in clinical trials of hair growth, with no clinical trials yet to elucidate its effects on cancer, including papillary thyroid cancer. The treatment, AT HAIR-FUL AA, represents this complex mixture, with notable levels of chlorogenic acid and procyanidin B2 at 400–600 and 60–100 μg/g, respectively [144]. It may be useful to evaluate the individual components of AFPE to identify the bioactive compounds that have the potential to specifically target PTC.

### 2.4. Oxidative Stress and Metabolism

#### 2.4.1. Resveratrol

Resveratrol (C_14_H_12_O_3_; 3,5,4′-trihydroxy-trans-stilbene) is a polyphenol that was detected in over 70 plant species, largely in grape skins and seeds [145]. Resveratrol has demonstrated bioactive effects, reported as cardioprotective, neuroprotective, antioxidant, anti-inflammatory, and anti-cancer, including colorectal, breast, liver, lung, ovarian, cervical, prostate cancer [146]. Thyroid cancer cell line Thr.C1-PI-33 treated with resveratrol at 10 and 50 μM had significantly decreased survival that was not seen in non-malignant fibroblast cells HFFF2 [147]. Treatment of Thr.C1-PI-33 with RAI and resveratrol demonstrated that resveratrol dose-dependently (5 μM, 10 μM, 50 μM) sensitized cells to RAI [147]. PTC cell lines BHP 2–7 and BHP 18–21 treated with resveratrol (1–10 μM) resulted in increased TP53 expression, TP53 serine phosphorylation, and c-Fos, c-Jun and p21 expression [148]. The TP53-dependent apoptosis in PTC induced by resveratrol was demonstrated to be mediated through the MAPK signal transduction pathway [148]. Involvement of the MAPK pathway was confirmed in BCPAP cells, in which resveratrol (10, 20, 40, 60, 80, and 100 μM) inhibited cell proliferation in a dose- and time-dependent manner. At 48- and 72-h treatment at the determined IC50s (18.7 and 56.8 μM, respectively) resveratrol acts by reducing BRAF and ERK expression, while increasing NIS expression [149]. Additionally, treatment of KTC-1 and TPC-1 cells with resveratrol dose-dependently inhibited proliferation (10, 50, 100, and 150 μM), and at 50 μM increasedG0/G1 cell cycle arrest, reduced clonogenicity, and induced apoptosis, evidenced by increased caspase-8, -9, and -3, Bax, and decreased B cell lymphoma-extra-large (Bcl-xl) and myeloid leukemia cell differentiation protein-1. Resveratrol at 50 μM reduced cell invasion and migration capability [150]. This was validated in a xenograft model whereby tumor growth rate was reduced, suggesting that resveratrol inhibits tumor growth and promotes survival in a mechanism involving reduced PI3K/Akt/mTOR pathway activation [150].

Resveratrol clinical trials in cancer include prostate, colorectal cancer, breast cancer, and multiple myeloma, whereby in multiple myeloma patients, adverse events occurred, however resveratrol was well-tolerated and safe in the other cancer clinical trials [151]. The major hurdles of resveratrol include its poor bioavailability and low potency past preclinical study. Nano-formulations decreased the IC50 of resveratrol by half in vitro [152,153], however this effect has yet to be elucidated in humans. Nano-formulated and free resveratrol efficacy will need to be determined in clinical trials before it is ready for clinical use. In normal rat thyrocytes, resveratrol decreased expression of thyroid differentiation makers thyroglobulin, thyroid peroxidase, TSHR, NK2 homebox-1, forkhead box E1, and PAX8 at 10 μM [154]. Thyroid differentiation markers are lost upon dedifferentiation and thyroid cancer progression, indicating a worse prognosis [155]. In vivo, Sprague-Dawley rats treated with 25 mg/kg of resveratrol saw increased size of the thyroid with normal serum TSH and thyroid hormone levels, therefore the authors caution that resveratrol may act as a thyroid disruptor and goitrogen [154].

#### 2.4.2. Rhodiolin

Rhodiolin (C_25_H_20_O_10_; (2R,3R)-6,8-dihydroxy-3-(4-hydroxy-3-methoxyphenyl)-2-(hydroxymethyl)-9-(4-hydroxyphenyl)-2,3-dihydropyrano[3,2-h][1,4]benzodioxin-7-one) is a flavonoid isolated from Rhodiola rosea thought to be critical for its therapeutic activity [156]. Investigation into its use in PTC demonstrated in vitro and in vivo inhibition of growth and the induction of apoptosis of TPC-1 and BCPAP cells, with no effect on immortalized normal thyroid cell line Nthy-ori 3-1 [156]. In vitro, rhodiolin dose-dependently decreased viability, clonogenicity, proliferation, and 3D organoid tumor volume (5, 10, 20, 40, and 80 μM). After 24 h of treatment at 10, 20, and 40 μM with rhodiolin, PTC cell lines reduced cell cycle progression evidenced by reductions in proliferating cell nuclear antigen., cyclin E, cdk4, and cdk2, and increased p21 and p27 expression. Apoptosis was further characterized by increased expression of total and cleaved caspase 3, Bax, total and cytoplasmic apoptosis inducible factor, total and cytoplasmic cytochrome C, and decreased Bcl-2. Transcriptomic and bioinformatic analysis implicated inhibition of the PI3k/Akt/mTOR signaling [156]. This signaling pathway was modulated through glucose-6-phosphate isomerase inhibition and blocking of glycolysis [156]. Glycolytic inhibition therefore is suggested to play a role in the mechanism of the anti-PTC effects of rhodiolin [156]. This was confirmed in vivo at 10 mg/kg and 25 mg/kg in a human PTC xenograft mouse model, whereby tumor growth was significantly decreased, and its ablation was promoted, while no adverse effects including hepatotoxicity or morphological abnormalities were observed upon hematoxylin and eosin staining. In vivo, rhodiolin was both efficacious and safe, however this has yet to be elucidated in human clinical trials of PTC.

### 2.5. Multi-Pathway Synergy

#### 2.5.1. EGCG

ECGC (C_22_H_18_O_11_; (–)-Epigallocatechin-3-gallate) is a polyphenolic compound that is the major catechin found in green tea [Camellia sinensis L. Ktze. (Theaceae)] dried fresh leaves [157]. EGCG has demonstrated antioxidant effects, improvements in cardiovascular health, enhancement of weight loss, protection of the skin from the damage caused by ionizing radiation, and anti-cancer effects, including colorectal, lung, breast, gastric, nasopharyngeal, pancreatic, hepatocellular, liver, ovarian, and prostate cancer [157,158]. Investigation of the effects of EGCG on PTC using TPC-1 cells has demonstrated the inhibition of cell proliferation, viability, cell cycle progression, migration, and invasion, with increased apoptosis in a dose-dependent manner (10, 25, 50, 100, and 200 μM) [159]. Treatment with EGCG resulted in the dose-dependent reduction in p-epidermal growth factor receptor (EGFR), H-RAS, p-RAF, p-MEK1/2, and p-ERK1/2 and promotion of Bax/Bcl-2 ratio, cleaved caspase-3 and cleaved PARP protein levels [159]. These data were validated in xenograft models in which tumor volume was reduced, angiogenesis was inhibited and apoptosis was induced [159]. This coincided with a dose-dependent increase in cleaved PARP positive cells and decrease in p-ERK1/2 positive cells [159]. The effect of EGCG on PTC was confirmed and extended in TPC-1 cells, demonstrating suppression of cell viability in 24 h at 10, 20, and 40 μM and in 48 h at 5, 10, 20, and 40 μM, with no effect on Nthy-ori 3-1 at the same doses. At the determined IC50 of 17.2 μM, ECGC resulted in the induction of apoptosis and cell cycle arrest in the S phase accompanied by the decreased cyclin A and cyclin-dependent kinase-2 expression [160]. Also observed were decreases in reactive oxygen species levels, upregulation of Bax expression, downregulation of Bcl-2 expression and increase in cytochrome C levels in the cytosol [160]. It was concluded that EGCG induced an autophagic response via the upregulation of the autophagy-related protein LC3-II and suppression of the AKT/mTOR signaling pathway [160]. Papillary thyroid cells, FB-2, also demonstrated a dose-dependent growth reduction by EGCG (10, 40, and 60 μM), which was associated with reduction in cyclin D1 and upregulation in p21 and p53, as well as the reduced phosphorylation of Akt and ERK1/2 [161]. Cell motility, migration, and invasion by gelatin zymography also decreased, which was accompanied by modulation of cell adhesion and actin cytoskeleton reorganization proteins, especially those involved in EMT, including increased E-cadherin and decreased Snail, Zeb, Twist, Vimentin, N-cadherin, and MMP-9 expression [161].

It has been suggested that oral administration of EGCG is lowly bioavailable in humans and decreases further when administered with food, however, other studies shown that specific nutrients and supplements may improve EGCG bioavailability [162]. Further, nanoparticles of EGCG dual-loaded with ascorbic acid showed enhanced therapeutic efficacy in Alzheimer’s models in vivo [163]. Green tea extract supplements come with risk of hepatotoxicity, including concentrated supplements of EGCG at 866 mg per day [164]. A randomized, double-blind, placebo-controlled trial demonstrated daily decaffeinated green tea extract at 856 mg EGCG for 16 weeks was well-tolerated and safe [165]. Clinical trials of EGCG include oral administration in idiopathic pulmonary fibrosis patients (ID NCT05195918) and for hepatocellular carcinoma chemoprevention (CATCH-B; ID NCT06015022). There are no clinical trials testing EGCG in PTC treatment.

#### 2.5.2. Silybin

Silybin (C_25_H_22_O_10_; (2S,3S)-3,5,7-trihydroxy-2-[(2S,3S)-3-(4-hydroxy-3-methoxyphenyl)-2-(hydroxymethyl)-2,3-dihydro-1,4-benzodioxin-6-yl]-2,3-dihydrochromen-4-one) is a polyphenolic flavonoid is obtained from the seeds of Silybum marianum (or milk thistle) and Cynara scolymus (or artichoke) and is the most biologically active component of silymarin [166]. Silybin has demonstrated antihepatotoxic, antioxidant, anti-inflammatory, and anti-cancer properties, including in oral, cervical, and gastric cancer [167]. Treatment of TPC-1 cells with silybin significantly reduced cell viability (100 μM) and 12-O-tetradecanoylphorbol-13-acetate (TPA)-induced cell migration and MMP-9 expression (50 μM) [168]. This coincided with the reduced phosphorylation of MEK and ERK, therefore supporting the idea that silybin suppresses TPA-induced cell migration and MMP-9 expression through the MAPK pathway in thyroid cancer cells [168]. Bioinformatic analysis to predict targets and an underlying mechanism of silybin treatment for PTC identified 489 molecular targets [169]. Functions and pathways impacted by silybin included PTC development, invasion, migration, and immunotherapy [169]. In vitro studies demonstrated that silybin treatment resulted in blockage of the Akt signaling pathway, reduction of the expression cyclin D1, N-cadherin, Vimentin, Snail, Slug, and programmed cell death ligand-1 expression while E-cadherin expression was significantly elevated [169]. These studies provide both theoretical and experimental scientific basis for the potential anti-cancer effects of silybin on PTC.

Silybin, like other natural compounds, has low oral bioavailability due to poor water solubility and poor intestinal absorption [166]. To increase its absorption and consequently therapeutic potential, largely for its use in liver diseases, phospholipid complex formulations, micellar systems, and water-soluble derivatives have been developed [170,171,172]. In a hepatocellular carcinoma, a Phase I study of silybin extract combined with phosphatidylcholine to increase absorption, called Siliphos, was investigated [173]. Siliphos treatment improved hepatic dysfunction in patients with advanced hepatocellular carcinoma upon dose-escalation 2, 4, 8, or 12 g per day for 12 weeks [173]. Silybin was detectable in serum and well-tolerated [173]. Clinical trials for silybin are largely focused on liver diseases, with clinical trials of some cancer types, but not PTC.

## 3. Future Directions

The natural compounds described have preclinical promise but lack clinical validation. Most of the findings are in vitro and in vivo studies that provide mechanistic insight without clinical correlations. The heterogeneity in PTC cell line genetic backgrounds may also play a critical role in how compounds lack translatability from in vitro to in vivo to humans. The downstream signaling resulting from BRAF mutations versus RET rearrangements can affect cell metabolism, oxidative stress, and hence confer sensitivity to compounds depending on the cell type used. Therefore, these cell lines may not successfully recapitulate the therapeutic potential of all natural compounds. Genetically diverse PTC models may better serve as representatives of natural compound efficacy. Future work also needs to be done to fully elucidate their mechanism of action in PTC, including the confirmation of some of the pathways involved that were speculated based on other cancers, as this will yield great benefit for development of novel drug candidates that can be used in combination with current therapeutic strategies. In silico network pharmacology, used by a few of the anti-PTC compounds, is very useful for understanding compound-target-disease relationships and extrapolating bioavailability and drug likeness, allowing for clinical prioritization of natural compounds.

These natural compounds can then be tested in vivo and in clinical trials of patients with PTC to validate efficacy, safety, and optimization of delivery. For some compounds, the dose of in vitro and in vivo efficacy needs to be assessed in humans to ensure the toxic dose is not reached for therapeutic efficacy. Once a mechanism is established and safety is confirmed, natural products are excellent clinical trial candidates. Some of the natural compounds mentioned are in clinical trials for anti-cancer effects, however PTC is not one of the targeted cancers. The overarching challenges of the described natural compounds include the issue of bioavailability. Novel drug delivery systems, including nanoparticle delivery, have begun to mitigate this issue [26]. However, more work needs to be done to justify the clinical readiness of these compounds that may have strong therapeutic potential.

## 4. Conclusions

**Figure 2:**
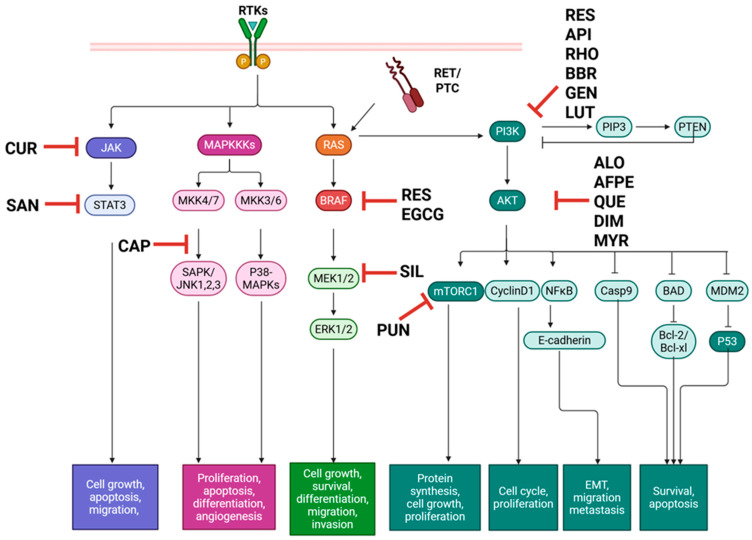
Natural compounds with therapeutic effects on PTC in vitro target major pathways MAPK, PI3K/Akt, and JAK/STAT, promoting apoptosis and preventing metastatic phenotypes. AFPE: *Annurca* Flesh Apple polyphenol extract, ALO: Aloperine, API: Apigenin, BAD: Bcl-2-associated agonist of cell death, BBR: Berberine, Bcl-XL: B-cell lymphoma extra-large, Bcl-2: B-cell lymphoma 2, BRAF: B-rapidly accelerated fibrosarcoma, Casp: Caspase, CAP: Capsaicin, CUR: Curcumin, DIM: 3,3′-Diindolylmethane, EGCG: Epigallocatechin gallate, ERK: Extracellular signal-regulated kinase, GEN: Genistein, JAK: Janus kinase, JNK: c-Jun N-terminal kinase, LUT: Luteolin; MAPK: Mitogen-activated protein kinase, MKK: MAPK kinase, MDM2: Mouse double minute 2, MEK: Mitogen-activated protein kinase kinase, mTOR: Mammalian target of rapamycin, MYR: Myrictein, NFκB: Nuclear Factor kappa-light-chain-enhancer of activated B cells, PIP3: Phosphatidylinositol 3,4,5-trisphosphate, PI3K/Akt: Phosphatidylinositol 3-kinase/protein kinase C, PTC: papillary thyroid carcinoma, PTEN: Phosphatase and tensin homolog, PUN: Punicalagin, p53: Tumor protein p53, QUE: Quercetin, RAS: Rat sarcoma virus, RES: Resveratrol, RET/PTC: Rearranged during transfection in papillary thyroid cancer, RHO: Rhodiolin, RTK: Receptor tyrosine kinase, SAN: Sanguinarine, SAPK: Stress-activated protein kinase, SIL: Silybin, and STAT: Signal transducer and activator of transcription. Created in BioRender. Geliebter, J. (2025) https://BioRender.com/coo9hrd (accessed on 9 August 2025).

The study of natural compounds in various diseases, including papillary thyroid cancer, has demonstrated significant efficacy (Table 3). In vitro and in silico, there has been a large body of evidence demonstrating the effects of these natural products on the ability of PTC cells to survive and metastasize (Figure 2).

## Figures and Tables

**Table 1:** Papillary thyroid cancer cell lines used for in vitro assessment of natural compounds. BRAF = B-rapidly accelerated fibrosarcoma, TP53 = tumor protein 53, and RET/PTC = rearranged ty-rosine kinase receptor in papillary thyroid cancer.

Cell Line	Known Mutation Status	References
K1	BRAFV600E^+^, TP53 ^mut^, PI3KCA ^mut^	[29,30,31]
TPC-1	RET/PTC^+^	[29,30,31]
BCPAP	BRAFV600E^+^, TP53 ^mut^	[29,30,31]
KTC-1	BRAFV600E^+^, TP53 ^mut^	[29,31]
BHP 2-7	RET/PTC^+^	[31]
BHP 18-21	BRAF^WT^, TP53 ^WT^	[32]
BHP 10-3	RET/PTC^+^	[31]
IHH-4	BRAFV600E^+^	[29]
NPA	BRAFV600E^+^, TP53 ^mut^	[31]
SNU-790	BRAFV600E^+^	[33]
FB-2	RET/PTC^+^	[34]

**Table 2:** Translational and therapeutic benefits of natural compounds for PTC.

Focus	Natural Compounds	Mechanism	Translational Benefit
**Overcoming RAI or Kinase Inhibitor Resistance**	Curcumin Quercetin Luteolin DIM Aloperine Genistein	Downregulation of STAT3, and survival signaling Resensitize cells to sorafenib	Potential combination adjuvants for advanced or recurrent PTC Reduce acquired resistance
**Apoptosis or Cell Cycle Arrest**	Berberine Myricetin Punicalagin Sanguinarine Aloperine Genistein	Activates p53, caspases, and Bax Suppresses BCL2 and surviving	May reduce recurrence and residual tumors after surgery
**Anti-Metastasis**	Luteolin Apigenin AFPE	Inhibits EMT and migration through PI3K/Akt and β-catenin suppression	May prevent recurrence and metastasis
**Oxidative stress and metabolism**	Resveratrol Rhodiolin Punicalagin	Regulate ROS, AMPK, and mitochondrial function	Improve tumor microenvironment and metabolic resilience

AFPE: Annurca Flesh Apple Polyphenol Extract, DIM: 3,3′-diindolylmethane, and EGCG: (–)-Epigallocatechin-3-gallate.

**Table 3:** The effect of natural compounds on papillary thyroid cancer cell phenotypes and pathways.

Compound	Herb/Source	Pathways	In Vitro/In Vivo Evidence	Effective Dose	Refs
**AFPE**	*Annurca* apple	PI3K/Akt	Reduction in cell viability Cell cycle arrest	250–1000 μM	[140]
**Aloperine**	*Sophora alopecuroides*	PI3K/Akt	Reduction of cell viability, clonogenicity Induction of autophagy	50–200+ μM	[82,83]
**Apigenin**	Parsley, chamomile, celery	PI3K/Akt	Inhibition of cell viability Promotion of autophagy and cell cycle arrest	20–30 μM	[134,135]
**Berberine**	*Berberis vulgaris*, *Coptis chinesis*, *Hydratis canadensis*	PI3K/Akt	Inhibition of cell growth Induction of apoptosis and G1 arrest Suppression of xenograft tumor growth	10–100 μM 100–200 mg/kg	[88,89,90]
**Capsaicin**	Chili peppers	PI3K/Akt	Reduction of viability, migration, invasion and adhesion	25–100 μM	[122,123]
**Curcumin**	*Curcuma longa*	PI3K/Akt JAK/STAT	Suppression of cell viability, proliferation, attachment, spreading, migration and invasion Induction of apoptosis	2.5–100 μM	[38,39,40]
**DIM**	Cruciferous vegetables	PI3K/Akt MAPK Estrogen signaling	Suppression of cell proliferation Induction of apoptosis	25–50 μM	[72]
**EGCG**	*Camellia sinensis*	MAPK PI3K/Akt	Inhibition of proliferation, viability, cell cycle progression, migration, and invasion Induction of autophagy	10–200 μM	[159,160,161]
**Genistein**	*Genista tinctoria*	PI3K/Akt	Reduction in proliferation Induction of cell cycle arrest	5–40 μg/mL	[116,117,118]
**Luteolin**	*Prunella vulgaris*	PI3k/Akt	Reduction of cell viability, proliferation, clonogenicity Induces cell cycle arrest Reduction of xenograft tumor growth Synergy with sorafenib	2.5–160 μM 50 mg/kg	[63,64]
**Myricetin**	Spinach, cauliflower, garlic, parsley, fennel	PI3K/Akt	Promotion of apoptosis	50–100 μM	[95]
**Punicalagin**	*Punica granatum*	MAPK	Reduction of cell viability and growth Induction of autophagy and senescence	50–100 μM	[102]
**Quercetin**	Capers, red onions and shallots, leafy greens	MAPK PI3K/Akt	Induction of apoptosis Reduction of cell proliferation, migration, and invasion Enhancement of sensitivity to kinase inhibitors	2.5–100 μM	[52,54,55,56]
**Resveratrol**	Grapes, skin and seeds	PI3K/Akt MAPK	Sensitization of cells to RAI, synergizing with chemotherapy Reduction in cell viability, inhibit migration Induction of apoptosis	5–100 μM	[147,148,149,150]
**Rhodiolin**	*Rhodiola rosea*	PI3K/Akt	Reduction in cell growth Induction of apoptosis Reduction in xenograft tumor growth	5–80 μM 25 mg/kg	[156]
**Sanguinarine**	*Sanguinaria canadensis*	JAK/STAT	Inhibition of cell growth and proliferation Induction of apoptosis and autophagy	2–8 μM	[112]
**Silybin**	*Silybum marianum*	PI3K/Akt MAPK	Reduction in migration and cell viability	100 μM	[168,169]

MAPK: Mitogen-activated protein kinase, PI3K/Akt: Phosphatidylinositol 3-kinase/protein kinase C, and RAI: Radioactive iodine.

## Data Availability

No new data were created or analyzed in this study. Data sharing is not applicable to this article.

## Abbreviations

The following abbreviations are used in this manuscript:

AFPE	*Annurca* Flesh Apple polyphenol extract
Akt	Protein kinase C
ATC	Anaplastic thyroid cancer
Bax	Bcl-2 associated X protein
BBR	Berberine
Bcl-XL	B-cell lymphoma extra-large
Bcl-2	B-cell lymphoma 2
BRAF	B-rapidly accelerated fibrosarcoma
cAMP	Cyclic adenosine monophosphate
CDK	Cyclin-dependent kinase
DIM	3,3′-Diindolylmethane
EGCG	Epigallocatechin gallate
EMT	Epithelial mesenchymal transition
ERK	Extracellular signal-regulated kinase
FGFR	Fibroblast growth factor receptor
HIF	Hypoxia inducible factor
HMOX1	Heme oxygenase 1
H_2_O_2_	Hydrogen peroxide
IL	Interleukin
JAK	Janus kinase
JNK	c-Jun N-terminal kinase
JUN	c-Jun proto-oncogene
KIT	c-Kit
LC3	Microtubule associated protein 1A/1B light chain
lncRNA	Long non-coding RNA
MAPK	Mitogen-activated protein kinase
MEK	Mitogen-activated protein kinase kinase
miRNA	Micro RNA
MMP	Matrix metalloprotease
mTOR	Mammalian target of rapamycin
NAG	Non-steroidal anti-inflammatory drug activated gene
NF-κB	Nuclear Factor kappa-light-chain-enhancer of activated B cells
NIS	Sodium iodide symporter
NRF2	Nuclear factor erythroid 2-related factor 2
NTRK	Neurotrophic tyrosine receptor kinase
PARP	Poly (ADP-Ribose)
PAX8/PPARγ	Paired box 8/peroxisome proliferator-activated receptor gamma-1
PDGFR	Platelet derived growth factor receptor
PI3K/Akt	Phosphatidylinositol 3-kinase/protein kinase C
PTC	Papillary thyroid cancer
PTEN	Phosphatase and tensin homolog
ROS	Reactive oxygen species
TC	Thyroid cancer
TERT	Telomerase reverse transcriptase
TGF	Transforming growth factor
TKI	Tyrosine kinase inhibitor
TPA	12-O-tetradecanoylphorbol-13-acetate
TPD	Thyroid proliferative disease
TP53	Tumor protein 53
TSHR	Thyroid stimulating hormone receptor
RAI	Radioactive iodine
RAS	Rat sarcoma virus
RET/PTC	Rearranged during transfection in papillary thyroid cancer
RR-PTC	Radioactive iodine refractory papillary thyroid cancer
RTK	Receptor tyrosine kinase
SASP	Senescence associated secretory phenotype
STAT	Signal transducer and activator of transcription
VEGF	Vascular endothelial growth factor
VEGFR	Vascular endothelial growth factor receptor
